# Pericementitis

**Published:** 1883-12

**Authors:** George A. Mills


ARTICLE VI.

### PERICEMENTITIS, ITS MANIFESTATIONS IN THE ORAL CAVITY, AND ITS SERIOUS EFFECTS UPON THE GENERAL HEALTH.—(Continued).

(Read, by invitation before the King's County Medical Society of Brooklyn, by DR. G. A. MILL'S, 169½ Columbia Heights, May 18, 1882)

### PERICEMENTUM PERICEMENTITIS AND ITS HISTOLOGICAL FEATURES.

To Dr. C. W. F. Bodecker, of New York City, we are indebted for the ablest paper upon this subject yet furnished us. It is the product of faithful observation made by actual work with the microscope in the laboratory of Carl Heitzman, and furnishes an understanding of this subject that makes the way quite plain. These articles can be found in thc *Cosmos*, and will be profitable reading to any who may feel an interest in the matter.

As it is not my purpose to environ this paper with any extended details upon the scientific aspect I will not enter largely upon its histological features, only noting the fact that they are comprised within both the myxomatous and fibrous connective tissue series, the former in the early life and the latter more advanced. This will account for the variable associations of discomforts between the early and later disturbances, the former attended with a less degree of pain ; and yet, under favorable circumstances, a greater rapidity of progress is made, for the reason that the one is endowed with less resistance than the other.

Pericementum has a connection of continuity both with myxomatous, or gum tissues and the periosteum. By being so allied to the cementum, a continued disturbance of the periosteum results in the complication of the disease, and the destructiveness not only of these but also the osseous portions forming the socket.

Pericementitis is an expression of a greater or less degree of debility resultant upon nerve degeneracy. It has a variety of phases, yet is more generally manifested at the peripheral margin of the gum. This is characterized sometimes by a slight tinge of congestion, changing the appearance from a normal, pinkish color to one of deeper red or purple, and at others to an anæmic or bloodless, or colorless appearance. This is followed by a detaching or relaxing the constriction of the membrane about the neck of the tooth. This is followed by the appearance of foreign substances, or by the absence of them, and an extended detachment of the tissues about the whole or a part of the neck of the tooth. The character of the inflammation—be it acute or otherwise, destructive or less so—is determined by the constitutional powers of resistance or the opposite. By this, I mean if there is a quantity of power to aid in the producing of the *quantum sufficit* of such equality of proportions to establish a normal status of repair; or if it be equal in one and deficient in another, there must necessarily be so overpowered as to result in an overplus of disturbances of territory, the weak succombing to the strong. This may be the awakening of the bond or bonds of energy in a normal degree, but yet being met by a bond of enfeebled affinity the results can only be destructive in a greater or less degree. Hence the necessity of something in the supply of nutriment that will be adapted to restore the enfeebled bond to its normal power of requisite affinity, so that the equality of waste and repair may be normally adjusted. This statement may or may not seem somewhat obscure, but my belief is that when the *modus-operandi* is understood of inflammatory action, it will then be made possible to accept this. That the time has, in a large measure, arrived for the physician of the future to establish the fact that his mission is of prevention rather than cure. We, in our investigations, look for exciting causes. To say that I am fully prepared to answer at this point would possibly seem quite like assumption. Many views and theories are advanced. Some attribute the cause to the presence of deposits of lime and their admixtures (so-called tartar). Some claim that these are the results of inflammatory action, and still others call them sanguinary deposits, or residum of the broken down tissues, blood, etc. Now, I cannot accept any of these views as the *cause* of the disease. The *cause* is *in esse*. By this, I may mean, or will so express as a general term, nerve degeneracy. This gives rise to the question of a definition for that. These questions all give rise to the acknowledgment of the impossibility of meeting all the points except upon the knowledge of the organization of tissues. As yet we know but in part, but we are accumulating a few postulates as the results of cultured discriminations as they are now being read from the works of nature through the microscope. And by this we may reasonably hope that the time is hastening when we wil be able to throw aside the curtain of mystery and reveal the deductions. While I have referred to the more general manifestations of this disease I have not noticed that there are many exciting causes, viz: Mechanical irritations, dead pulps, alveolar abscess, crowded conditions of the teeth, accumulations of foreign substances, etc., etc. Not a few cases manifest a peculiar phase, noticed particularly associated with the exhibit of a recession of gum-tissue and not any inflammatory action apparent. These are dominated atrophy of the gum. It is thought by many to be caused by friction of the brush. While this might, under some circumstances, facilitate the loss of tissue, yet it is far from being the cause. This phase is seen at points where the brush would fail to have any such effect, viz: not only on the labial and buccal, but on the lingual and palatal surfaces of the teeth; and I would add that in many of these cases there is no perceptible presence of deposits of lime.

You will notice that I have, in passing, pointed out the manifestations of the disease in their mildest expressions. Starting out with the familar adage that prevention is better than cure, it becomes of decided importance to emphasize familiarity with incipient stages, for if intelligence is active at this stage, we have under control the staying of its future destructiveness. The serious effects of this disease upon the general health are so well known to those who have familiarized themselves by an earnest and vigorous study of its workings, that it would be a crime to sit in silence and not proclaim the agonies associated. I am satisfied that large numbers are being cut off from their pilgrimage here prematurely, while thousands are dragging out a drooping existence of lassitude, depression and inanition directly and indirectly traceable to this disease. Perhaps I cannot do better than to state a case which will serve the purpose of demonstrating the many.

In the fall of 1878, Dr. Mason, Sr., President of the Long Island Medical College, called at my office and consulted me abont a patient of his who was in a wretched and rapid state of decline of health. He said he and his son had exhausted their remedies upon this patient, and he, having seen my articles published in Bronchure, had become impressed that possibly this patient was a victim to the disease I had called his attention to. Several dentists had been consulted, but not with encouragement, excepting the extraction of the teeth. The patient came into my hands. She was about thirty-eight years of age, strong, nervous-billious temperament, married. I found her with a dry, parched skin, feeeble pulse, loss of appetite, depressed —sadly—sleepless, nausea on waking in the morning, and great loss of nerve energy. She had twenty-nine beautifully formed teeth, so loose that she had not been able to masticate with any power for a long time—some two years I think. From every socket was exuding a fetid discharge and very copious, so much so that she was obliged to place two large napkins under the side of her face to receive this flow at night while she slept. Now this case did not prove to be one of suppurative pericementitis alone; it had involved the osseous formations, and that portion involving the sockets of the teeth was more or less destroyed. This case proved in treatment the necessity of surgical attention in the direction claimed by Dr. Riggs, as I have described. It also proved that it had not been developed altogether during the time she had become cognizant of it, but circumstances of such severity had fastened upon her and so checked the activities of her organization that it was left with an enfeebled power to cope with the disorder already present. The patient being so highly organized, her sufferings were of the acute order, and played great havoc among the distribution of the sensory nerves. The result of the treatment in this case, both surgical and constitutional, brought the patient again into the sphere of activity and usefulness. To use her own words, " she was as good as new." The exciting cause of the rapid decline of this patient was a *terriffic mental grief.* I could detail numerous cases that have come under my notice during the last eight years, particularly where a variety of associate disorders had become complicated. I do not need to pursue the enumeration of these facts. You are familar with many instances where the progress of disorders frequently reveal before unknown ones, resulting in prolonged distress, and not uncommonly, loss of life.

And now I do not think I need to go further, for I do not doubt that the intelligent mind will grasp a proper measure of the truths to which I have called your attention, and which are readily demonstrated in the oral cavities of ninety per cent. of the people, in a greater or less degree of activity. I will not leave you with the impression that the specialty I represent is wholly alive, or in a large sense cognizant of the nature of the destruction that is traveling madly over their every-day practice. The importance of surgical ability, as I said in my introductory, is becoming to be felt as the advance step necessary to bring this branch to its needed elevation for usefulness. As yet dentistry, as practiced by the masses, can claim to be no more than Webster gives them credit for : " one who repairs teeth." To my understanding dentistry has a distinctly separate line from oral surgery, and will, I predict, in the near future be so estimated by intelligent people of discernment.

Gentlemen, it is from your ranks that much aid can come to assist those that are zealously devoting their energies to raise this special feature—oral surgery—to its sphere of greater usefulness in the alleviation of human suffering. And if you have been convinced, by what I have brought to your attention, of a conception of its importance, then I will have not spoken in vain.

The resume of this paper leads me to say this: that the revival of interest in this subject, by being brought up under a new feature, has proved aggressive, and by the controversy, interrogation by thought and action has given additional knowledge. It can no longer be viewed as a trival matter, for the fact is established that it is a specific disease, exhibiting specified manifestations and amenable to treatment, under the same limitations as all diseases; also that trained perception and cultured discrimination, gained by concentrated investigations and practice, produces a grade of skill above that of the novice. Further, that the serious import of this subject to the public cannot be emphasized too strongly, for they cannot know too early that which is *first* our duty to be acquainted and impressed with, and in proportion as we come into possession of this knowledge, and are made conscious of its purpose in our hands, we will impress them by the alleviation of their sufferings. "He that hungers and thirsts for knowledge, will, in the giving of it, unconsciously use it as a blessing and a joy to many."—*Texas Dental Journal.*

